# Random Search Walks Inside Absorbing Annuli

**DOI:** 10.3390/e27070758

**Published:** 2025-07-15

**Authors:** Anderson S. Bibiano-Filho, Jandson F. O. de Freitas, Marcos G. E. da Luz, Gandhimohan M. Viswanathan, Ernesto P. Raposo

**Affiliations:** 1 Laboratório de Física Teórica e Computacional, Departamento de Física, Universidade Federal de Pernambuco, Recife 50670-901, PE, Brazil; anderson.bibianofilho@ufpe.br (A.S.B.-F.); ernesto.raposo@ufpe.br (E.P.R.); 2 Departamento de Física, Universidade Federal de Viçosa, Viçosa 36570-900, MG, Brazil; jandson.freitas@ufv.br; 3 Departamento de Física, Universidade Federal do Paraná, Curitiba 81531-980, PR, Brazil; luz@fisica.ufpr.br; 4 Department of Physics and National Institute of Science and Technology of Complex Systems, Federal University of Rio Grande do Norte, Natal 59078-970, RN, Brazil

**Keywords:** random search, Lévy walks, physics of foraging

## Abstract

We revisit the problem of random search walks in the two-dimensional (2D) space between concentric absorbing annuli, in which a searcher performs random steps until finding either the inner or the outer ring. By considering step lengths drawn from a power-law distribution, we obtain the exact analytical result for the search efficiency η in the ballistic limit, as well as an approximate expression for η in the regime of searches starting far away from both rings, and the scaling behavior of η for very small initial distances to the inner ring. Our numerical results show good overall agreement with the theoretical findings. We also analyze numerically the absorbing probabilities related to the encounter of the inner and outer rings and the associated Shannon entropy. The power-law exponent marking the crossing of such probabilities (equiprobability) and the maximum entropy condition grows logarithmically with the starting distance. Random search walks inside absorbing annuli are relevant, since they represent a mean-field approach to conventional random searches in 2D, which is still an open problem with important applications in various fields.

## 1. Introduction

Random searches constitute a class of statistical physics problems [1,2,3,4,5,6] in which a random walker takes steps in a D-dimensional search space while seeking target sites whose locations are partially or fully unknown. Random search models usually consider the length and direction of the random steps as drawn from probability distributions, and a step length is eventually truncated by finding a target. Applications of random searches exist in various fields, from information technology [7,8] and sporozoites looking for hotspots in blood vessels in the malaria infection process [9] to adaptive exploration control of autonomous flying robots [10] and animal foraging [5,6], to name a few.

An important statistical measure in random search problems is the search efficiency η, which is defined [11] as the number of targets found along the search walk divided by the total distance traveled or by the total walk duration. In particular, the optimization of η as a function of the search parameters is key to practical applications. For example, an animal that forages with low efficiency may starve to death [11].

Despite continuous efforts over the last decades, some central issues in the random search problem still remain unsolved. For instance, the exact formal expression of the search efficiency in 1D search space has been derived in terms of an integral operator, with the kernel given by the probability density of the step lengths [12,13]. In contrast, the exact analytical form of the efficiency in 2D and higher-dimensional spaces is still an open issue [1,2].

In this context, we have recently introduced [14] the problem of random searches inside concentric absorbing annuli, in which a searcher performs random steps in the 2D region between the rings until finding either the inner or the outer annulus. As the searcher can never go beyond the external ring, this problem represents a mean-field approach to the conventional random searches in 2D, i.e., all steps with lengths larger than the diameter of the outer annulus certainly end up truncated by the finding of a ring. In this sense, we have recently considered the random searches inside absorbing annuli to approach the question of the optimization of the search efficiency in the case that the searcher takes step lengths from a Lévy distribution [14]. The results point to a maximum η for the Lévy index α=1 when the searcher starts quite close to the inner ring and the outer ring is very distant, helping to settle a recent debate [15,16,17].

Here, we investigate random searches inside absorbing annuli with a focus on step lengths drawn from a power-law density. It is well known that the asymptotic limit of Lévy α-stable distributions with Lévy stability index α∈(0,2) is given by the power-law density with exponent α+1 (for α=2, the Lévy distribution recovers the Gaussian) [18,19]. By leveraging the fact that power-law distributions are generally more amenable to calculations than Lévy distributions, we obtain the exact search efficiency in the α→0 ballistic limit, as well as an approximate expression for η in the regime of searches starting very far from both rings, and we rederive the scaling behavior of η for quite small initial distances to the inner ring. We also analyze the absorbing probabilities related to the finding of the inner and outer rings and the associated Shannon entropy. We study the values of the exponent α that set the equiprobability and maximum entropy conditions as a function of the searcher’s starting distance. Our numerical results display good overall agreement with the theoretical predictions.

**Figure 1:**
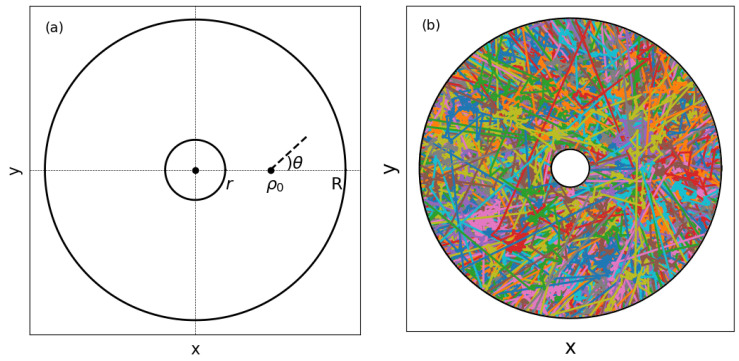
(**a**) The search space of random walks inside concentric absorbing annuli is the 2D region between the inner and outer rings of radii *r* and *R*, respectively. The searcher starts from a distance $\rho_{0}$ to the center of the rings and takes an angle θ. (**b**) Illustration of a set of $N_{w}=10^{4}$ walks (depicted in different colors) with step lengths taken from the power-law P(ℓ) for α=1, R=7, r=1, $\rho_{0}=R/2$, and $\ell_{0}=10^{-2}$.

The problem of random search walks inside absorbing annuli is worth investigating for a number of reasons. On the empirical side, we shall mention a rather illustrative example. A recent study [20] of the scale-free movement patterns of a species of termites has been conducted in a diversity of arenas, including the case of two concentric petri dishes, which is essentially the same geometry of the present work; see Figure 1a. From the biochemical point of view, interactions between termites and the environment or other termites are short-ranged and involve mechanical contact and chemical recognition so that, e.g., when a termite reaches either the inner or outer annulus, the individual goes on a brief exploration and then ignores the petri border [20]. The authors fitted the step lengths of the termites to a power-law distribution of exponent α+1, as given in Equations (1) and (2) below. Interestingly, they found exponents in the range of α∈[1.5,2.0], depending on the number of individuals in the experiment (we note that the exponent α is denoted as μ in Ref. [20]). Another aspect is that the original foraging problem can be analyzed indirectly by proxy through characterizing the first passage time processes in the annuli geometry. Indeed, the first passage time can be thought as the basic building block, representing the finding of successive targets in the random search for many targets. In the present case, the inner annulus represents the previously found target site in the foraging problem, while the outer ring replaces all other targets in a mean-field approach. This type of reasoning can be applied, e.g., to a patchy environment. In fact, when the resources are distributed in patches, the forager tends to migrate between these distinct rich regions. So, in our model, the inner ring would describe the patch just left by the searcher. By their turn, all other distant patches that can be visited could be effectively represented by the outer ring. So, our annuli landscape can be considered a mean-field approximation for patchy foraging. Finally, from a theoretical perspective, the problem with the annuli geometry is amenable to some elucidating analytic calculations that otherwise could be difficult to derive in the conventional random search problem (or foraging problem) in 2D. For example, the constraint imposed by the presence of the external ring allows for the exact calculation of the search (foraging) efficiency in the ballistic limit, as shown below. Moreover, an approximate result for the efficiency is also derived here in the scarce foraging regime in which the searcher starts very far away from all target sites.

This article is organized as follows. In Section 2, we define the random search model, and in Section 3, we obtain some analytical results in specific search regimes. The numerical results are presented and discussed in Section 4. Lastly, final remarks and conclusions are left to Section 5.

## 2. Random Search Model Inside Absorbing Annuli

Figure 1a illustrates the search space comprising the 2D region between the inner and outer concentric absorbing rings of radii *r* and *R*, respectively. The searcher leaves from a distance $\rho_{0}$ to the center of the annuli. Each random step is characterized by a length ℓ>0 and an angle θ∈[0,2π) defined, e.g., with respect to the horizontal positive semi-axis. Random search models usually consider *ℓ* and θ as independent variables, with values drawn from probability distributions p(ℓ) and ω(θ), respectively. Here, we take the angles as uniformly distributed, ω(θ)=1/(2π), and focus on the search behavior as the parameters that set p(ℓ) are varied.

The sum of independent and identically distributed random variables displays two statistical attractors, namely, the Gaussian and Lévy α-stable distributions, driven by the central limit theorem (CLT) and generalized CLT, respectively [18,19]. While the variance in the Gaussian density is finite, giving rise to normal diffusive dynamics of the random walker, the second moment of Lévy distributions diverges for Lévy index α∈(0,2), leading to superdiffusion (in the limit case α=2, the Gaussian density and normal diffusion are recovered). Thus, in many practical applications of Lévy or Lévy-type processes, such as in biology [21,22,23,24], fluid mechanics [25], and photonics [26,27,28], the superdiffusive dynamics are limited in time and/or spatial scales, and the CLT behavior is retrieved for very large numbers of steps [29,30].

In this sense, power-law (Pareto) distributions of step lengths represent a versatile model for the statistical dynamic behavior of the random searcher, which can shift from the CLT to the generalized CLT by only changing a single parameter. Indeed, by writing the probability density of step lengths in the power-law form, we have

(1) $$P\left(\ell \right)=\frac{\left(\mu -1\right)}{\ell_{0}}{\left(\frac{\ell_{0}}{\ell }\right)}^{\mu },\;\ell \geq \ell_{0},$$

and P(ℓ)=0 for $0<\ell <\ell_{0}$, with $\ell_{0}$ as the minimum step length. Thus, P(ℓ) is governed by the CLT if μ≥3, and corresponds to the asymptotic large-*ℓ* limit of symmetric Lévy α-stable distributions for

(2) μ=α+1,withα∈(0,2),

and for a suitable choice of $\ell_{0}$ in terms of α and the scale parameter of the Lévy distributions [18,19]. Another advantage of working with power-law distributions of step lengths is the fact that the calculations are generally easier than with Lévy distributions. Indeed, closed-form expressions for symmetric Lévy distributions in terms of simple mathematical functions are known only for α=1 (Cauchy–Lorentz) and the limit case α=2 (Gaussian) [18,19,31].

## 3. Some Analytical Results in Limit Search Regimes

In this section, we analytically address some random search regimes using the power-law distribution of step lengths given in Equations (1) and (2).

### 3.1. Ballistic Limit

We first consider the α→0 ballistic limit of extremely large rectilinear steps, in which the searcher’s first move always finds either the inner or the outer ring.

If the searcher leaves from a distance $\rho_{0}$ to the center of the annuli and takes the step direction making an angle θ defined in the upper half of the plane, as shown in Figure 1a, then in the ballistic regime, the outer (inner) ring is found for $0\leq \theta <\bar{\theta }$ ($\bar{\theta }\leq \theta \leq \pi$), where $\bar{\theta }=\pi -\sin^{-1}\left(r/\rho_{0}\right)$. We note by symmetry that similar conditions can also be written for angles in the lower half of the plane.

Upon the encounter of a ring, the step is truncated so that, by the cosine law, its effective truncated length is

(3) $$\ell_{s}\left(\theta \right)=-\rho_{0}\cos\left(\theta \right)+\zeta {\left[\xi^{2}-\rho_{0}^{2}\sin^{2}\left(\theta \right)\right]}^{1/2},$$

in which ζ=1 (ζ=−1) and ξ=R (ξ=r) when the outer (inner) ring is reached. The mean step length averaged over the uniform density of angles, ω(θ)=1/(2π), is exactly expressed as

(4) $$\langle \ell_{s}\rangle =\frac{R}{\pi }E\left(\bar{\theta },\rho_{0}^{2}/R^{2}\right)+\frac{r}{\pi }\left[E\left(\bar{\theta },\rho_{0}^{2}/r^{2}\right)-E\left(\pi ,\rho_{0}^{2}/r^{2}\right)\right],$$

where E(φ,k) denotes the incomplete elliptic integral of the second kind [32]. Thus, in the α→0 one-step ballistic limit, the search efficiency is simply given by $\eta =1/\langle \ell_{s}\rangle$. We shall probe Equation (4) numerically in Section 4.

### 3.2. Searches Starting Very Far from Both Annuli

We now turn to the case in which the searcher starts from a point very distant from both rings, with R≫r. For example, in the symmetric start, one has $\rho_{0}=(R+r)/2\approx R/2$ so that $r\ll \rho_{0}\ll R$. In the foraging problem, this condition resembles the destructive searches in the very low target density regime (corresponding to taking the limit R→∞ in the annuli search problem) [11].

Let us denote by $P_{n}^{\mathrm{surv}}\left(\rho_{0}\right)$ the searcher’s survival probability after *n* steps, i.e., the probability that neither ring has been found up to *n* moves, for searches with starting distance $\rho_{0}$. So, the probability of finding one of the annuli exactly at the *n*-th step is $P_{n}\left(\rho_{0}\right)=P_{n-1}^{\mathrm{surv}}-P_{n}^{\mathrm{surv}}$. As mentioned, in the search problem inside absorbing rings, the searcher stops moving once any annulus is reached so that it cannot go beyond the external ring. This process bears some resemblance to random searches on a finite 1D interval with absorbing boundaries, in which the searcher cannot jump over any of the two boundary sites. In both cases, in the large-*n* limit, the asymptotic survival probability decays exponentially with the number of steps (or with the time in a continuous time search walk) [33,34] so that $P_{n}^{\mathrm{surv}}\approx Ae^{-\gamma n}$, with *A* and γ positive parameters. So in this regime, we write

(5) $$P_{n}\approx Ae^{-\gamma (n-1)}p,$$

with $p=1-e^{-\gamma }$ denoting the probability that one of the rings is reached at the *n*-th last single step.

On the other hand, the mean search length is given by

(6) $$\langle L\rangle \left(\rho_{0}\right)=\frac{\sum_{n=1}^{\infty }P_{n}\langle L_{n}\rangle }{\sum_{n=1}^{\infty }P_{n}},$$

where $\langle L_{n}\rangle$ is the mean length of walks that finds one of the rings after *n* steps. By combining $\langle L_{n}\rangle \approx n\langle \ell \rangle$ with Equations (5) and (6), we obtain

(7) $$\langle L\rangle \approx \frac{\langle \ell \rangle }{p}.$$

We note that walks that effectively reach the large-*n* regime with the exponential asymptotics given by Equation (5) are favored by initial conditions in which the searcher starts very distant from both rings, $r\ll \rho_{0}\ll R$, and by values of α away from the ballistic limit; otherwise, the search should likely end by the finding of a ring before the large-*n* regime of Equation (5) sets in.

The probability *p* in Equation (7) is given by

(8) $$p=\frac{1}{\pi }\int_{0}^{\pi }d\theta \int_{\ell_{s}\left(\theta \right)}^{\infty }P\left(\ell \right)d\ell ,$$

where the integral in *ℓ* accounts for the probability of single steps of length equal or larger than $\ell_{s}\left(\theta \right)$ that reach one of the annuli (we recall that steps of length $>\ell_{s}$ are truncated by the encounter of a ring), with $\ell_{s}$ in Equation (3) and $\rho_{0}$ replaced by the starting position ${\bar{\rho }}_{0}$ of the last step. Furthermore, the integral in θ in Equation (8) averages over the uniformly distributed angles. Similarly, we write

(9) $$\langle \ell \rangle =\frac{1}{\pi }\int_{0}^{\pi }d\theta \left[\int_{0}^{\ell_{s}\left(\theta \right)}\ell P\left(\ell \right)d\ell +\ell_{s}\left(\theta \right)\int_{\ell_{s}\left(\theta \right)}^{\infty }P\left(\ell \right)d\ell \right],$$

with the truncated steps taken into account in the second integral. Finally, considering Equations (7) and (9) for the power-law P(ℓ), Equation (1) leads to the efficiency in this regime, with the searcher starting very far from both rings:

(10) $$\eta \approx \frac{\left(1-\alpha )I(\alpha \right)}{I\left(\alpha -1\right)-\pi \alpha \ell_{0}^{1-\alpha }},$$

where

(11) I(α)=1π2α+1ρ¯0αsinπα2aα 2F1α2,α2;α+1;u−4αbαρ¯02R2−ρ¯02αF12−α2,−α2;1−α;u+sin−1rρ¯0(ρ¯0−r)−α−(R+ρ¯0)−α,

with the notations $a_{\alpha }=\Gamma \left((1-\alpha )/2\right)\Gamma \left(\alpha /2\right)$, $b_{\alpha }=\Gamma \left(-\alpha /2\right)\Gamma \left((\alpha +1)/2\right)$, $u=1-R^{2}/{\bar{\rho }}_{0}^{2}$, and the Gaussian hypergeometric function F12 [32]. In Section 4, we compare the result (10) with numerical simulations.

### 3.3. Searches Starting Very near the Inner Annulus

We now consider the regime in which the searcher starts very close to the inner ring. We review the scaling analysis of the search efficiency as a function of the initial distance to the inner annulus, in the limit this distance is very small and the searcher never gets too far away along the search [11,14]. While the former condition implies $\rho_{0}\to r$, or in terms of a dimensionless parameter,

(12) $$\delta \equiv (\rho_{0}-r)/r\to 0,$$

the latter limit can be achieved by further setting $\ell_{0}\to 0$ in the power-law density. In the foraging literature, this regime is often referred to as the non-destructive search [11]. As mentioned, we additionally consider the corresponding low-target density limit, with a very far outer ring with R→∞.

When the searcher starts quite close to the inner annulus (δ→0) and never goes too far in the case of $\ell_{0}\to 0$, then this ring effectively looks like a “flat wall”. So, the search dynamics can be approximated by a 1D description, and the rigorous theory of the Riesz operator on a finite 1D interval with absorbing boundaries becomes applicable [12,15]. Moreover, since the probability of finding the very distant outer ring (R→∞) is vanishingly small, then the search much probably ends by the encounter of the inner annulus. Therefore, this set of limits $(\delta \to 0,\;\ell_{0}\to 0,\;$R→∞) ensures that the searcher never wanders too far beyond some region of radius ∼ar before being absorbed, where 1≲a≪R/r. In this case, the scaling behavior with δ of the mean number 〈n〉 of steps to find the inner ring approximately follows the 1D result on a finite absorbing interval [12], but with the distance between the boundary sites in 1D replaced by ar:

(13) $$\langle n\rangle ∼{\left[\frac{r^{2}\delta \left(a-\delta \right)}{\ell_{0}^{2}}\right]}^{\alpha /2},$$

apart from some multiplicative constant not dependent on δ. By the same reasoning, the mean step length is

(14) $$\langle \ell \rangle ∼\ell_{0}\left[b{\left(\frac{\delta r}{\ell_{0}}\right)}^{1-\alpha }+1\right],$$

with *b* being also independent of δ. Thus, by writing η≈1/(〈n〉〈ℓ〉) in the limit δ→0, we find the scaling behavior of the efficiency in this regime, with the searcher starting very close to the inner ring:

(15) $$\eta ∼\left\{\begin{array}{cc} \;\delta^{-1+\alpha /2} & ,\;\;\alpha >1,\\ \;\delta^{-\alpha /2} & ,\;\;\alpha <1. \end{array}\right.$$

We note in this case that η reaches a maximum for $\alpha =\alpha_{max}=1$. Interestingly, the scaling result above, valid in the triple limit $\delta \to 0,\;\ell_{0}\to 0,\;R\to \infty$, coincides with the behavior of both 1D [12] and 2D [15] foraging efficiencies in the non-destructive search regime [11].

On the other hand, if any of these three limits is not achieved, we show numerically in Section 4 that a progressive downshift of the value of $\alpha_{\max}$ in the range $\alpha_{\max}\in \left(0,1\right)$ generally takes place. In particular, in the regime considered in Section 3.2, with the searcher starting from a point very far from both rings, a ballistic strategy with $\alpha_{\max}\to 0$ leads to the maximum efficiency, as we see below.

## 4. Results and Discussion

We now present the numerical simulation results of random search walks inside the absorbing annuli. In the numerical procedure, the searcher starts from an initial distance $\rho_{0}$ to the center of the rings, where $r<\rho_{0}<R$. Before each move, the searcher’s step direction is set by an angle θ∈[0,2π) drawn from the uniform density, ω(θ)=1/(2π), and the move length *ℓ* is taken from the power-law density, as shown in Equation (1). If one of the rings is reached along the course of a given move, so the step is truncated and the distance traversed in that search realization is recorded for averaging. The search then restarts with the searcher placed at the same initial distance $\rho_{0}$ to the center.

Two termination criteria for the simulations can be chosen with statistically equivalent results. We can either fix a large total distance $L_{\mathrm{tot}}$ traveled by the searcher and count the number $N_{w}\gg 1$ of times that any of the annuli is found along $L_{\mathrm{tot}}$ (i.e., the number of statistically independent search walks along $L_{\mathrm{tot}}$), or alternatively, we fix a large $N_{w}$ and sum up the total distance traveled, $L_{\mathrm{tot}}=\sum_{j=1}^{N_{w}}L_{j}$, where $L_{j}$ is the distance traversed in the *j*-th search walk. In both cases, the search efficiency is given by [11]

(16) $$\eta =\frac{N_{w}}{L_{\mathrm{tot}}}=\frac{1}{\langle L\rangle },$$

where we recall that 〈L〉 is the mean search length, with the average taken over the $N_{w}$ search realizations. We comment that the presence of the outer ring yields 〈L〉 to be well defined for the power-law distribution of step lengths with any value of α, since all steps larger than the diameter of the external ring end up truncated. In contrast, 〈L〉 diverges for α∈(0,1] in free space with no boundary constraints.

In addition to the choice of the termination criterion ($L_{\mathrm{tot}}$ or $N_{w}$), the simulation parameters are the radii of the inner (*r*) and outer (*R*) annuli, the starting distance $\rho_{0}$—or equivalently, the relative initial distance δ to the inner ring—Equation (12), the power-law exponent α, and the minimum step length $\ell_{0}$. In all results below, we set r=1. To illustrate the search process, Figure 1b displays a collection of $N_{w}=10^{4}$ walks (depicted in different colors), with the step lengths taken from the power-law P(ℓ), using α=1, R=7, $\rho_{0}=R/2$, and $\ell_{0}=10^{-2}$. Interestingly, for this set of parameters, the superposition of these walks nearly fills up the space between the rings.

**Figure 2:**
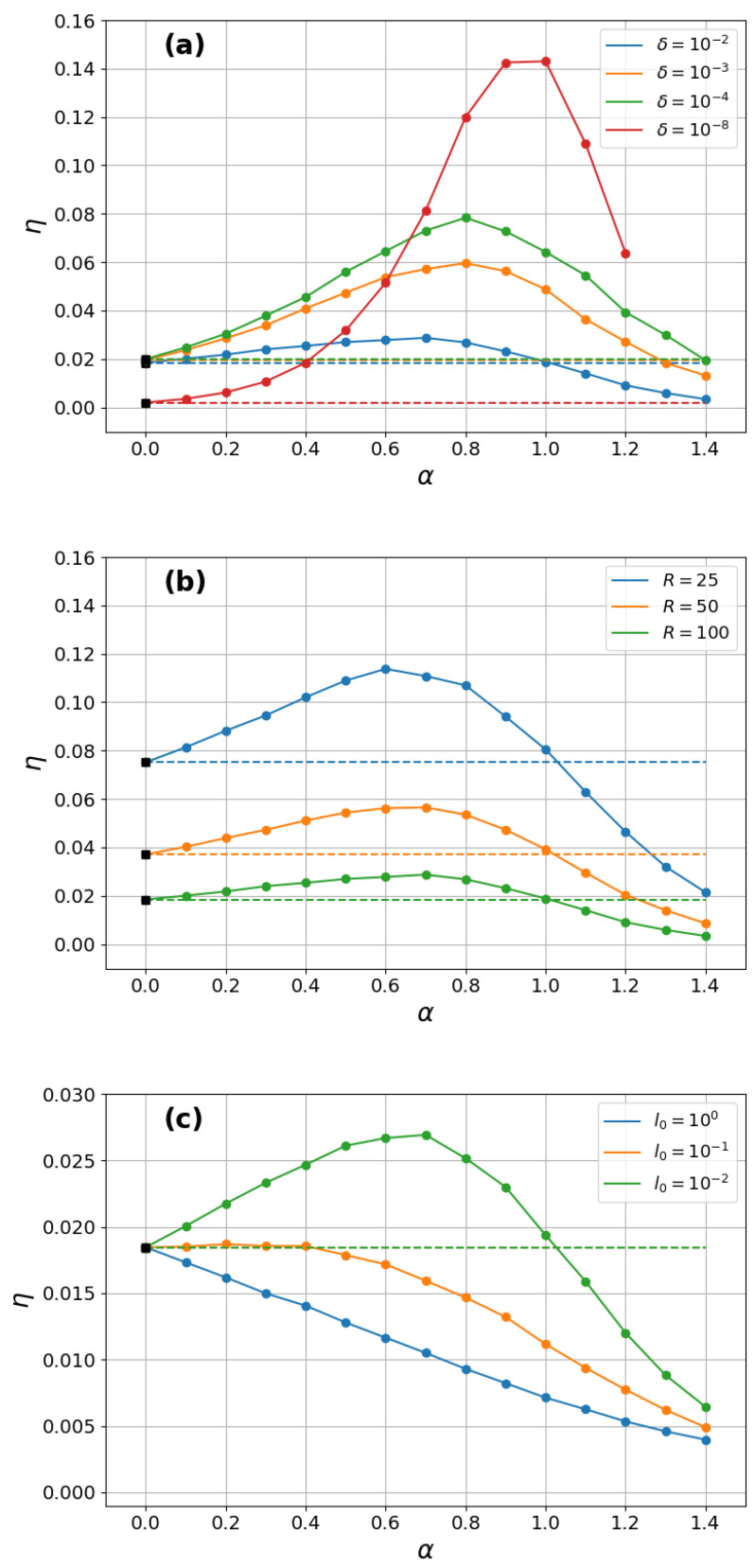
Search efficiency η as a function of α for several sets of parameters in the regime of searches starting very near the inner ring. The fixed parameters were (**a**) $R=10^{2}$ and $\ell_{0}=10^{-3}$; (**b**) $\delta =10^{-2}$ and $\ell_{0}=10^{-3}$; and (**c**) $\delta =10^{-3}$ and $R=10^{2}$. In the triple limit $\delta \to 0,\ell_{0}\to 0,R\to \infty$, η is maximum for $\alpha =\alpha_{\max}=1$, which is in agreement with the red curve in (a) (we have multiplied η in this curve by the factor 1/10 for better visualization). In all cases, we note a nice match of the numerical data to the exact analytical result in the ballistic limit, $\eta =1/\langle \ell_{s}\rangle$, with $\langle \ell_{s}\rangle$ in Equation (4), depicted by black squares indicated by the horizontal dashed lines as α→0.

We first analyze the searches that start very near the inner ring. Figure 2 shows the efficiency η as a function of α for several sets of parameters and termination criterion $N_{w}=10^{5}$. As discussed in Section 3.3, when the searcher starts very close to the inner ring and never gets too far away, the efficiency η displays a maximum for $\alpha =\alpha_{\max}=1$ in the triple limit $\delta \to 0,\;\ell_{0}\to 0,\;R\to \infty$. This result agrees with the red curve shown in Figure 2a for $\delta =10^{-8},\ell_{0}=10^{-3},$ and $R=10^{2}$.

If, on the other hand, any of these three parameters gets progressively far from the respective limits, we observe in Figure 2a–c a gradual downshift of $\alpha_{\max}$ in the range $\alpha_{\max}\in \left(0,1\right)$. For instance, in Figure 2a, we notice a decrease in $\alpha_{\max}$ toward the value $\alpha_{\max}\approx 0.7$ upon an increase in the relative starting distance from $\delta =10^{-8}$ to $\delta =10^{-2}$, while keeping the other parameters fixed. Similarly, a reduction in $\alpha_{\max}$ is also observed by either decreasing *R* or increasing $\ell_{0}$; see Figure 2b,c, respectively. Interestingly, we note in Figure 2c that for large enough $\ell_{0}$ values, the ballistic strategy $\alpha_{\max}\to 0$ is the one with the maximum η. In all cases, we observe a nice match of the numerical data to the exact analytical result in the ballistic limit, $\eta =1/\langle \ell_{s}\rangle$, with $\langle \ell_{s}\rangle$ in Equation (4), which are depicted in black squares in Figure 2a–c and indicated by the horizontal dashed lines as α→0.

We present in Ref. [14] a plot similar to Figure 2 but for the Lévy P(ℓ) density. We note, however, that simulations using the Lévy density of step lengths are much more time-consuming due to the numerical algorithm for sampling Lévy-distributed random numbers [35,36]. Thus, as the results for power-law and Lévy P(ℓ) distributions are qualitatively similar, we have focused on the former in this work. However, we stress that this is the only plot in [14] with an analogue in the present work, i.e., all other figures and analyses displayed below do not have any counterpart in Ref. [14].

**Figure 3:**
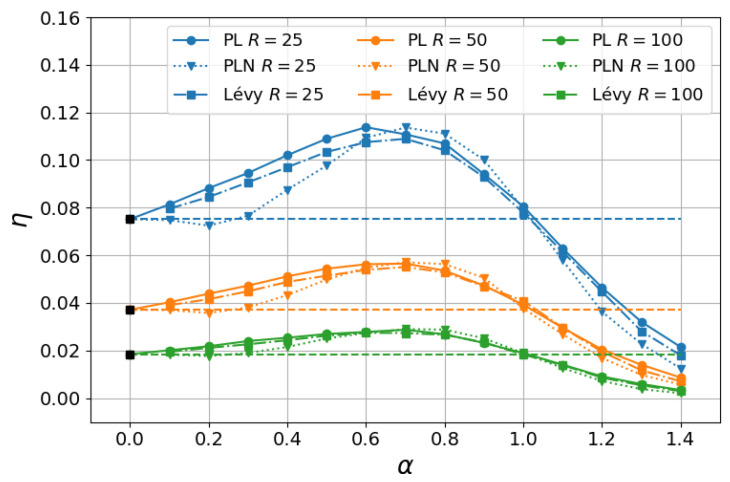
Search efficiency η as a function of α for fixed $\delta =10^{-2}$ and $\ell_{0}=10^{-3}$ and choices of the external radius R=25 (blue), R=50 (orange), and R=100 (green). For comparison, we display results with the step lengths drawn from the power-law distribution given in Equations (1) and (2) (PL shown as circles), Lévy α-stable distribution (color squares), and sum of N=10 power-law-distributed variables (PLN shown as triangles). The horizontal dashed lines indicate the exact analytical result in the α→0 ballistic limit (black squares).

In this sense, we show in Figure 3 a comparison between the numerical results when the step lengths are drawn from power-law and Lévy α-stable distributions. Parameters were chosen as in Figure 2b, with fixed $\delta =10^{-2}$ and $\ell_{0}=10^{-3}$, and choices of the external radius R=25 (blue), R=50 (orange), and R=100 (green). Two aspects can be observed in Figure 3. First, as mentioned, the power-law distribution given in Equations (1) and (2) corresponds to the dominant term in the large-*ℓ* expansion of the symmetric Lévy α-stable distribution [18,19]. In fact, we notice in Figure 3 that the results for the power-law (PL shown as circles) and Lévy (squares) distributions are close in the case of the largest external radius R=100 (green curves), and they became progressively different as smaller *R* were considered, as expected. Another interesting aspect is comparing these results with those in which each step length results from a sum of *N* power-law-distributed random variables (PLN shown as triangles). More precisely, in this case, each step length is given by $\ell =\sum_{i=1}^{N}x_{i}/N$, where each $x_{i}$ is drawn from the power-law probability density $P(x_{i})$ given in Equations (1) and (2). As commented above, the generalized CLT states [18,19] that the P(ℓ) of the PLN case converges in the limit N→∞ to a Lévy α-stable distribution if $\left\{x_{i}\right\}$ is a set of independent and identically distributed variables and α∈(0,2) (for α=2, the Gaussian P(ℓ) and CLT are retrieved). In other words, noises arising from power-law probability distribution functions are not stable in the context of the CLT, in contrast to the α-stable noises, though their statistics present similar long-range behavior. We note in Figure 3 that the results for Lévy and PLN are similar for the largest R=100, though some differences arose for R=25, mainly in the regime α<1 in which the first momentum of the Lévy and PLN distributions diverge. A more comprehensive study of how these results approach as much larger *N* are considered, as stated by the generalized CLT, is left for future work.

**Figure 4:**
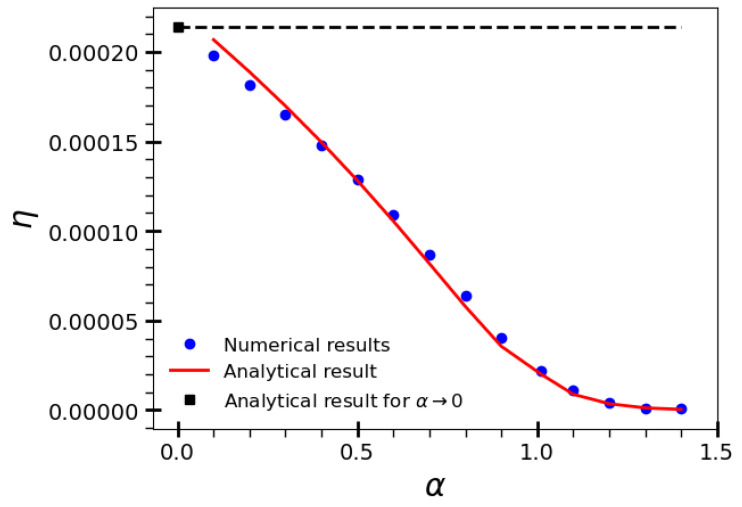
Search efficiency η as a function of α for $R=5\times 10^{3}$, $\rho_{0}=R/2$, and $\ell_{0}=10^{-3}$ in the regime of searches starting very far from both rings. The approximate result of Equation (10) is shown in red line, with good overall agreement to the numerical data depicted in blue circles. We also note a nice agreement of the numerical trend with the α→0 ballistic result given in Equation (4) (black square indicated by the dashed line).

We now turn to the regime considered in Section 3.2 of searches starting very far from both rings, with $r\ll \rho_{0}\ll R$ and large *R*. Figure 4 shows in blue circles the numerical results for $R=5\times 10^{3}$, $\rho_{0}=R/2$, and $\ell_{0}=10^{-3}$. The termination criteria were $L_{tot}=10^{9}$, $10^{8},$ and $10^{7}$ for α∈[0.1,0.5],[0.6,0.8], and [0.9,1.4], respectively. The approximate result of Equation (10) is shown in the red line, with good overall agreement to the numerical data. We recall that ${\bar{\rho }}_{0}$ in Equation (11) is the starting distance of the last step that ends up by finding one of the rings. In this regime, due to the search symmetry in the wide free space between the absorbing annuli, one can conveniently write ${\bar{\rho }}_{0}=\nu R$, with ν not much different from 1/2, at least for searches with α-values not close to the ballistic limit. In fact, the good agreement in Figure 4 was achieved for ν=0.62. We also notice that the match of the blue circles and red line is not so good as the ballistic regime, α→0, is approached. Furthermore, we observe as well a nice agreement of the numerical trend with the α→0 ballistic result given in Equation (4), shown in black square.

**Figure 5:**
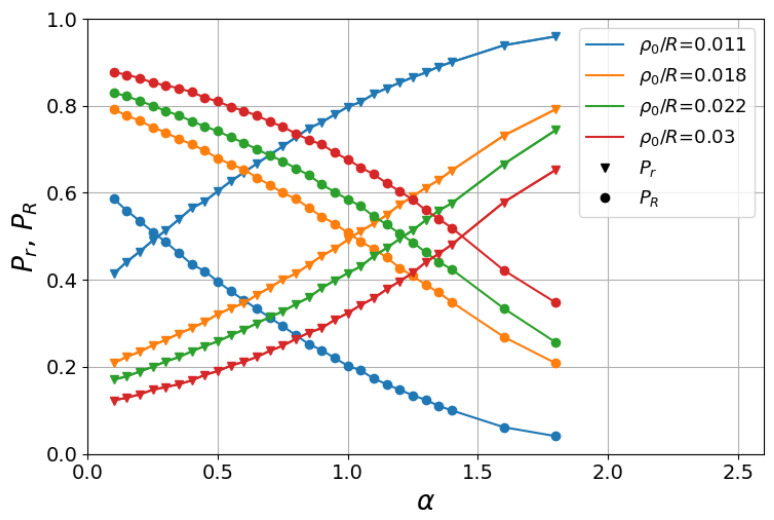
Absorbing probabilities, $P_{r}$ and $P_{R}$, of finding the inner and outer rings, respectively, as a function of α in the regime of searches starting near the inner ring. Numerical results ($P_{r}$: triangles; $P_{R}$: circles) are displayed for several values of $\rho_{0}/R$, $R=10^{2}$, and $\ell_{0}=10^{-3}$. The equiprobability condition, $P_{r}=P_{R}=1/2$, is achieved for values $\alpha =\alpha^{*}\left(\rho_{0}/R\right)$, marking the crossing of curves of same color. Lines are only guides to the eye.

We next see in Figure 5 the absorbing probabilities, $P_{r}$ and $P_{R}$, that the searcher finds the inner and outer rings, respectively, as a function of α and for several values of $\rho_{0}/R$. We considered searches starting much closer to the inner than to the outer annulus, where $r≲\rho_{0}\ll R$. We also note that $P_{r}+P_{R}=1$, since each search ended up by the encounter of one of the rings. In this analysis, we set $R=10^{2}$, $\ell_{0}=10^{-3}$, and $N_{w}=10^{5}$.

We observe in Figure 5 that for each $\rho_{0}/R$, there exists a value of α for which the finding of the inner or outer ring is equiprobable, where $P_{r}=P_{R}=1/2$. Interestingly, we remark that this probability crossover is also present in the 2D foraging problem, but not in the 1D foraging scenario [37]. This fact can be understood as follows. First, in 1D foraging, if the searcher takes a step with the minimum length necessary to find one of the boundary sites, it will certainly reach it, since jumps over boundary targets without detection are forbidden. In this sense, due to the left–right symmetry of the 1D search, the initially closest target is always found with higher probability, thus preventing the equity of absorbing probabilities from occurring in 1D.

On the other hand, in search walks inside absorbing annuli and in 2D foraging as well, if the searcher does not take a step direction within the correct angle range, then it will miss the inner ring (or the closest target site in the foraging problem), even if the step length is larger than the usual Euclidean distance to this ring. In particular, in the large-*ℓ* limit of small α, if a wrong direction is initially taken, the searcher will likely head to a great distance from the starting point already in the very first move. It is thus clear that, in contrast to the 1D case, depending on the values of α and $\rho_{0}/R$, the probability $P_{r}$ of finding the initially close inner ring may not always be higher than the probability $P_{R}$ of encountering the distant outer ring, and hence the equiprobability of $P_{r}$ and $P_{R}$ can occur in 2D searches.

To illustrate this fact, let us denote by $\alpha^{*}\left(\rho_{0}/R\right)$ the value of α as a function of $\rho_{0}/R$ for which $P_{r}=P_{R}=1/2$. We notice in Figure 5 that $P_{r}>P_{R}$ for $\alpha >\alpha^{*}$, while $P_{r}<P_{R}$ for $\alpha <\alpha^{*}$. This means that searchers starting from the same initial distance in the range $r≲\rho_{0}\ll R$ find the close inner ring more likely by generally performing smaller steps, favored by larger values of α. Indeed, as argued, low values of α typically lead to large steps that, if not taken in the proper direction, may leave the searcher very far away from the inner ring, thus increasing the chance of finding the initially more distant outer ring.

In a complementary view, the results above can also be read from the analysis of the associated Shannon entropy [38],

(17) $$S\left(\rho_{0},\alpha \right)=-P_{r}\log_{2}P_{r}-P_{R}\log_{2}P_{R}.$$

**Figure 6:**
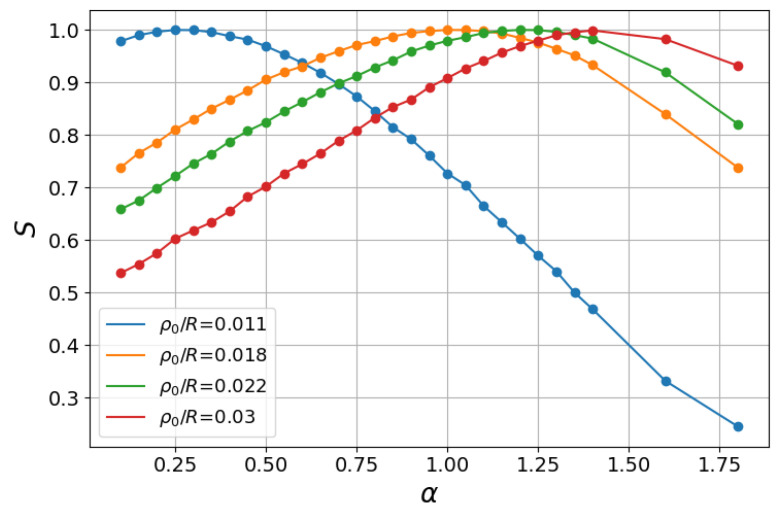
Shannon entropy *S*, Equation (17), associated with the absorbing probabilities, $P_{r}$ and $P_{R}$, as a function of α. Parameters as in Figure 5. The equiprobability condition, $P_{r}=P_{R}=1/2$, implies $S_{\max}=1$ at values $\alpha =\alpha^{*}\left(\rho_{0}/R\right)$.

Shannon entropy (17) has the maximum value, $S_{\max}=1$, for the equiprobability condition, $P_{r}=P_{R}=1/2$, i.e., along the curve $\alpha^{*}\left(\rho_{0}/R\right)$. Figure 6 displays *S* as a function of α for the same values of $\rho_{0}/R$ and parameters of Figure 5. For each $\rho_{0}/R$, we observe the presence of the maximum $S_{\max}=1$ at $\alpha =\alpha^{*}$ separating two regions, namely, a regime with increasing *S* for $\alpha <\alpha^{*}$ and a subsequent one with decreasing *S* for $\alpha >\alpha^{*}$. However, two limiting search strategies can be also identified in Figure 6 that led to regimes in which the Shannon entropy was monotonically decreasing $\left(\alpha^{*}\to 0\right)$ or monotonically increasing $\left(\alpha^{*}\to 2\right)$. Their associated mechanisms can be explained as follows.

On the one hand, when the searcher starts quite close to the inner annulus, $\rho_{0}\to r=1$, a very large (truncated) first step in the α→0 ballistic strategy yields the encounter of the inner ring if the step direction lies in the range θ∈(π/2,3π/2) or the far away outer ring in the complementary interval. In the case of the uniform density of angles, this implies that $P_{r}=P_{R}=1/2$ and $S_{\max}=1$ for $\rho_{0}\to r=1$ and $\alpha^{*}\to 0$, leading to a monotonically decreasing *S* for α>0. In Figure 6, this result can be approximately inferred from the blue curve with $\rho_{0}/R=0.011$, in which the maximum *S* is reached for a low $\alpha^{*}$.

On the other hand, in the opposite regime of steps typically very small, i.e., in the limit α→2 with $\ell_{0}\to 0$, the finding of the inner or outer annulus is equiprobable when the searcher is initially nearly equidistant from both annuli, $\rho_{0}\approx \left(R+r\right)/2\approx R/2$, with large *R*. In this case, one has $P_{r}=P_{R}=1/2$ and $S_{\max}=1$ for $\rho_{0}\approx R/2$ and $\alpha^{*}\to 2$, leading to a monotonically increasing *S* for 0<α<2. We note in the curves of Figure 6 that the trend for a monotonically increasing entropy in this interval emerged as higher values of $\rho_{0}/R$ were considered, being almost already achieved for $\rho_{0}/R=0.03$.

**Figure 7:**
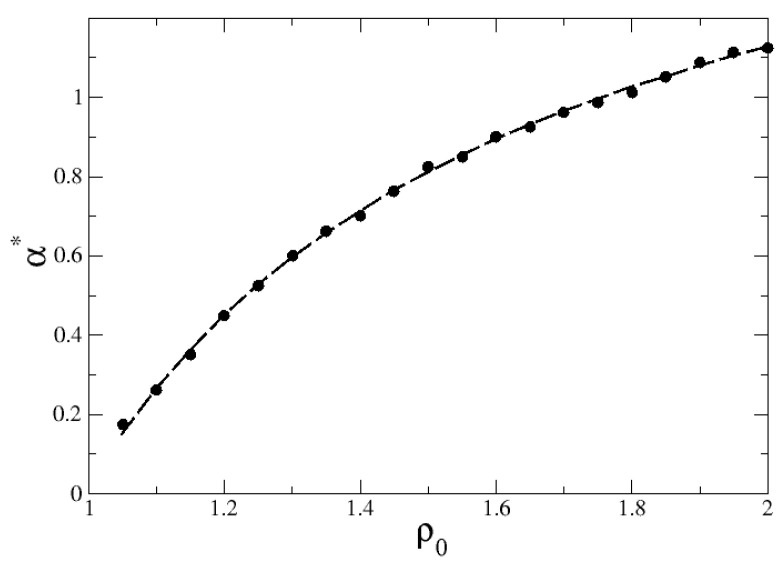
Values $\alpha =\alpha^{*}$ at which Shannon entropy (17) is maximum, $S_{\max}=1$, and the equiprobability condition, $P_{r}=P_{R}=1/2$, is achieved as a function of $\rho_{0}$. Parameters as in Figure 5. Numerical data are depicted in circles, and the dashed line is the best fit to the logarithmic growth in Equation (18), with $a_{0}=3.23$, $a_{1}=5.12$, $a_{2}=1.37$, and $a_{3}=2.82$.

Finally, Figure 7 shows via circles the numerical values of $\alpha^{*}$ as a function of $\rho_{0}$, along which the maximum Shannon entropy, $S_{\max}=1$, and the equiprobability condition, $P_{r}=P_{R}=1/2$, having been achieved. The parameters were set as in Figure 5. A theoretical fit of this curve is possible in the regime where the searcher starts very close to the inner ring, $\rho_{0}\to r=1$, and its minimum step length $\ell_{0}\to 0$, so that the probability of finding the distant outer ring is vanishingly small in the large-*R* limit. As mentioned, in this case, the search dynamics can be approximated by a 1D description. In the context of searches on a 1D finite interval with absorbing boundaries, the probability of finding the initially very far boundary site is given by [13] $P_{L}\left(x_{0}\right)=f\left(x_{0}/L,\alpha \right){\left(x_{0}/L\right)}^{\alpha /2}$, where $x_{0}$ is the initial distance to the closest site, *L* is the length of the 1D search space, and *f* is some function of $x_{0}/L$ and α. When $x_{0}\to 0$, its asymptotic limit reads as $P_{L}\left(x_{0}\right)∼{\left(x_{0}/L\right)}^{\alpha /2}$. In the present case, this should correspond to $P_{R}\left(\rho_{0}\right)∼{\left[\left(\rho_{0}-r\right)/R\right]}^{\alpha /2}$. Considering the equiprobability condition, $P_{R}=1/2$ with $\alpha =\alpha^{*}$, and writing $P_{R}\left(\rho_{0}\right)=a_{0}{\left[a_{1}\rho_{0}-a_{2}\right]}^{\alpha /2}$, we obtain

(18) $$\alpha^{*}\approx -\frac{2\ln(2a_{0})}{\ln(a_{1}\rho_{0}-a_{2})}+a_{3},$$

where we have introduced the quantity $a_{3}$ given in terms of the other parameters so that the limit α→0 when $\rho_{0}\to r=1$ is retrieved. Figure 7 displays through a dashed line the good fit of the numerical data to Equation (18).

## 5. Final Remarks and Conclusions

In conclusion, in this work, we have studied the problem of random search walks inside absorbing annuli with a power-law distribution of step lengths. On the analytical side, we have obtained the exact expression of the search efficiency η in the α→0 ballistic limit, as well as an approximate result for η in the regime of searches starting very far from both rings and the scaling behavior of η for quite small starting distances from the inner ring. We have also provided numerical results for the efficiency as a function of the power-law exponent α for several sets of parameters comprising the mentioned regimes, with good overall agreement with the analytical findings.

We have also analyzed the absorbing probabilities related to the encounter of the inner and outer rings and the associated Shannon entropy results. In particular, we have studied the exponent value $\alpha =\alpha^{*}$ as a function of the searcher’s starting distance, which marks the crossing of such probabilities (equiprobability) and the maximum entropy condition.

We emphasize that our results significantly advance and extend those of Ref. [14]. Indeed, on the analytical side, both the exact calculation of the search efficiency in the ballistic limit and the approximated computation of the efficiency in the regime in which the searcher starts very far away from the annuli constitute new findings. Furthermore, the numerically discussed trends of the absorbing probabilities and Shannon entropy, not analyzed in [14], as well as the identification that the power-law exponent marking the equiprobability and maximum entropy conditions grows logarithmically with the starting distance, represent uncovered characteristics of this kind of search landscape.

Finally, we comment on the relevance of the present findings in two different contexts. From a more mathematical point of view, random search walks inside absorbing annuli (and D-dimensional hyperspheres as well) represent a mean-field approach to random searches in two (and higher) spatial dimensions, representing a problem which from the formal point of view is still open. So, our results can be a starting point for new theoretical approaches trying to solve it.

The present study also relates to the potential practical ecological interest in defining in the foraging problem an entropy function such as that given in Equation (17). Assuming that a random searcher (forager) can look for $k=1,2,\ldots ,N_{t}$ types of targets [39], the entropy function could read as $S=-\sum_{k}P_{k}\log_{N_{t}}\left(P_{k}\right)$. Since *S* is sensitive to the spatial distribution of targets and search space dimensionality, fractal environments and, more significantly, fragmented landscapes [40,41] could be eventually inferred by computing *S* once one knows the frequency $f_{k}$ (leading to $P_{k}$) that a forager captures each target type *k* (e.g., $f_{k}$ could in principle be determined empirically). Identifying the degree of fragmentation of a given habitat represents a fundamental concern in conservation biology, with important consequences for biodiversity and even species survival in a given region [42,43,44]. In this sense, *S* could be a proper quantifier to identify the quality/degradation of a habitat. Currently, we are working on these ideas, and the results will be published in due course.

## Data Availability

The data presented in this study are available upon request from the corresponding author.
